# Cortical synaptic and dendritic spine abnormalities in a presymptomatic TDP-43 model of amyotrophic lateral sclerosis

**DOI:** 10.1038/srep37968

**Published:** 2016-11-29

**Authors:** Matthew J. Fogarty, Paul M. Klenowski, John D. Lee, Joy R. Drieberg-Thompson, Selena E. Bartlett, Shyuan T. Ngo, Massimo A. Hilliard, Mark C. Bellingham, Peter G. Noakes

**Affiliations:** 1 School of Biomedical Sciences, The University of Queensland, Brisbane, QLD, Australia; 2 Translational Research Institute, Queensland University of Technology, Brisbane, QLD, Australia; 3 Clem Jones Centre for Ageing Dementia Research, Queensland Brain Institute, The University of Queensland, Brisbane, QLD, Australia; 4 University of Queensland Centre for Clinical Research, The University of Queensland, Brisbane, QLD, Australia; 5 Department of Neurology, Royal Brisbane & Women’s Hospital, Brisbane, QLD, Australia

## Abstract

Layer V pyramidal neurons (LVPNs) within the motor cortex integrate sensory cues and co-ordinate voluntary control of motor output. In amyotrophic lateral sclerosis (ALS) LVPNs and spinal motor neurons degenerate. The pathogenesis of neural degeneration is unknown in ALS; 10% of cases have a genetic cause, whereas 90% are sporadic, with most of the latter showing TDP-43 inclusions. Clinical and experimental evidence implicate excitotoxicity as a prime aetiological candidate. Using patch clamp and dye-filling techniques in brain slices, combined with high-resolution confocal microscopy, we report increased excitatory synaptic inputs and dendritic spine densities in early presymptomatic mice carrying a TDP-43^Q331K^ mutation. These findings demonstrate substantive alterations in the motor cortex neural network, long before an overt degenerative phenotype has been reported. We conclude that increased excitatory neurotransmission is a common pathophysiology amongst differing genetic cases of ALS and may be of relevance to the 95% of sporadic ALS cases that exhibit TDP-43 inclusions.

Amyotrophic lateral sclerosis (ALS) is the most common motor neuron disease and is clinically characterized by the death of corticospinal motor neurons (MNs) in the cortex and alpha MNs in the brainstem and spinal cord (upper and lower MNs, respectively), leading inexorably to muscle weakness progressing to death within 3–5 years after diagnosis^1^,^2^. ALS occurs in both sporadic (90–95% of patients) and familial (5–10% of patients with a hereditary genetic mutation) forms, with its heterogeneous clinical symptoms being highly conserved between the two forms^1^.

The discovery of TAR DNA-binding protein 43 (TDP-43) inclusion bodies in the cytoplasm, nucleus and neurites of sporadic and familial ALS^3^,^4^,^5^ implicates common pathological processes, as over 95% of all patients (both sporadic and familial) exhibit TDP-43 aggregation as part of their pathology^6^,^7^. Clinical investigations have identified cortical electrical hyperexcitablity abnormalities in patients^8^,^9^,^10^,^11^,^12^ and that glutamate-mediated functional and structural alterations in both upper and lower MNs may be present in sporadic as well as familial ALS^2^,^8^,^13^,^14^. In addition, postmortem studies of brains from ALS patients, demonstrate that layer V pyramidal neurons (LVPNs) exhibit dendritic abnormalities consistent with degeneration^15^.

Taken together, these findings in humans and those from validated animal models^16^,^17^,^18^ pose the intriguing question as to whether or not TDP-43 mutations may contribute to altered neuronal synaptic activity and structure, particularly at stages prior to neuronal loss. Although ALS is an adult-onset disease, there is a lengthy pre-symptomatic period during which glutamate excitotoxicity may be particularly important^19^,^20^. For example, in superoxide dismutase 1 (SOD1) rodent models of ALS, excessive glutamatergic neurotransmission and associated dendritic and dendritic spine structural abnormalities have been observed in upper and lower MNs long before significant MN death^16^,^17^,^18^,^21^,^22^. This may be of key importance in the motor cortex, where genetic suppression of abnormal SOD1 expression is able to delay disease onset while prolonging both upper and lower MN survival^23^.

Here, we aimed to determine whether functional synaptic, dendritic and dendritic spine abnormalities, occur in LVPNs from the motor cortex of presymptomatic TDP-43^Q331K^ mice, at an age when cortical growth and connectivity has plateaued - post-natal (P) days 26–35^24^,^25^ - but before the onset of neurodegeneration^26^. Using patch clamp and dye-filling methods in acute brain slices, combined with high resolution confocal imaging, we report the first cellular electrophysiological recordings in any TDP-43 rodent model that show increased spontaneous excitatory synaptic transmission and increased dendritic spine density. Our findings suggest that excessive glutamatergic neurotransmission to LVPNs of the motor cortex is a common feature of ALS, occurring before neuronal death and other symptoms.

## Results

### TDP-43^Q331K^ causes increased excitatory synaptic neurotransmission to LVPNs of the motor cortex

**Figure 1:**
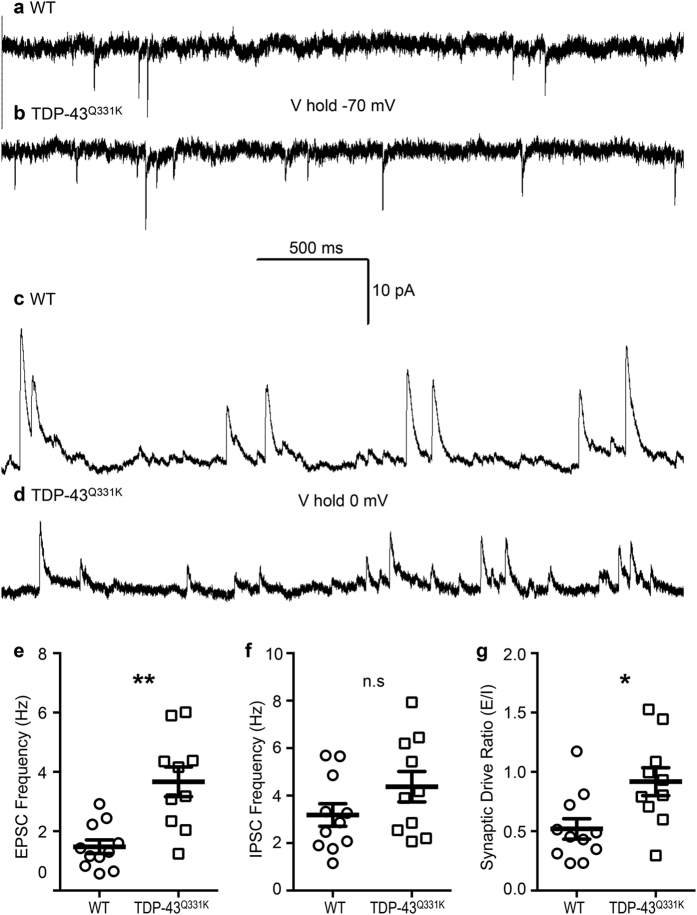
Increased excitatory synaptic activity on LVPNs within the motor cortex in TDP-43^Q331K^ mutant animals. (**a**,**b**) Show representative traces of EPSCs (inward currents) at a holding voltage of −70 mV in WT and TDP-43^Q331K^ LVPNs, respectively. (**c**,**d**) Show representative traces of IPSCs (outward currents) at a holding voltage of 0 mV in WT and TDP-43^Q331K^ LVPNs, respectively. (**e**) Shows a scatterplot with mean ± s.e.m. of increased EPSC frequency (Hz) in TDP-43^Q331K^ LVPNs (*n* = 10) compared to WT age- and litter-matched controls (*n* = 11), **P < 0.01, Mann Whitney test. (**f**) Shows a scatterplot with mean ± s.e.m. of unchanged IPSC frequency (Hz) in TDP-43^Q331K^ LVPNs (*n* = 10) compared to WT age- and litter-matched controls (*n* = 11), Students unpaired two-tailed *t* test. (**g**) Shows a scatterplot with mean ± s.e.m. of increased synaptic drive ratio (EPSC frequency/IPSC frequency) in TDP-43^Q331K^ LVPNs (*n* = 10) compared to WT age- and litter-matched controls (*n* = 11), *P < 0.05, students unpaired two-tailed *t* test.

Synaptic dysfunction is common across many neurodegenerative disorders^27^ and increased glutamatergic synaptic neurotransmission has been observed in upper and lower MNs from mouse models of ALS^16^,^17^,^18^. To determine the effect of the TDP-43^Q331K^ mutation on functional synaptic activity, we analyzed spontaneous EPSCs (excitatory post synaptic currents) and IPSCs (inhibitory post synaptic currents) recorded from LVPNs in the motor cortex (Table 1). Excitatory neurotransmission frequency, detected as EPSC, was increased by 148% in TDP-43^Q331K^ compared to WT (**P = 0.001, Table 1, Fig. 1). There was no difference in LVPN EPSC amplitude, rise time, or half-width between WT and TDP-43^Q331K^ mice (Table 1). Inhibitory neurotransmission, detected as IPSC, was comparable between the two genotypes (frequency, amplitude, rise time and half-width; Table 1, Fig. 1). The synaptic drive ratio (the ratio of EPSC to IPSC frequency) was increased by 77% in TDP-43^Q331K^ mice when compared to WT (*P = 0.01, Table 1, Fig. 1). These measurements were not influenced by changes in input resistence between genotypes, which were not significantly different (WT: 201 ± 55 MΩ, *n* = 6; TDP-43^Q331K^: 184 ± 29, *n* = 5, *P* = 0.77).

Taken together, our patch-clamp data suggest that TDP-43^Q331K^ mice exhibit a functionally higher level of excitatory inputs onto LVPNs of the motor cortex, without any compensatory change in the level of inhibitory inputs. This may underpin perturbations in the network and firing activity of the motor cortex and may lead to glutamate-mediated excitotoxicity, long thought to be one of the main aetiological candidates in ALS and other neurodegenerative disorders^2^,^19^,^20^,^27^,^28^,^29^. These functional alterations in excitatory synaptic neurotransmitter inputs onto LVPNs are also likely to alter dendritic morphology, which contribes to the integration and funnelling of these inputs to the soma^30^. We therefore investigated the dendritic morphology of LVPNs in the motor cortex receiving these altered synaptic inputs.

### Increased excitatory synaptic inputs onto motor cortical LVPNs of TDP-43^Q331K^ mice results in increased apical and basal dendritic spine densities, but does not change dendritic length

**Figure 2:**
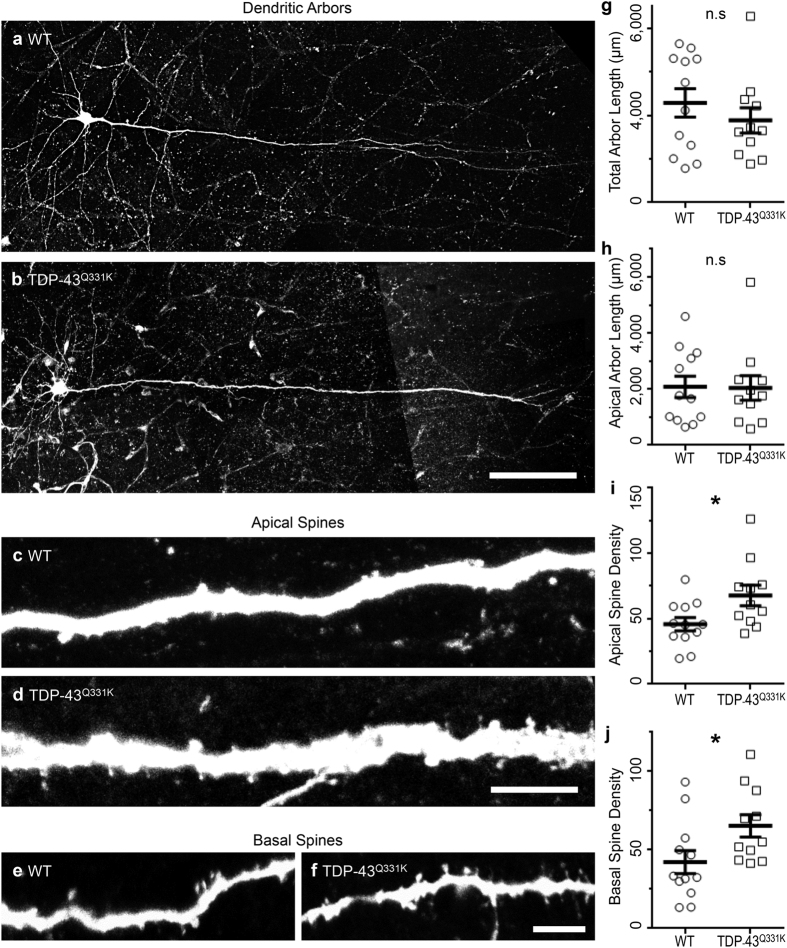
Stable neuronal arbor length and increased apical and basal dendritic spine densities in LVPNs within the motor cortex of TDP-43^Q331K^ mutant animals. (**a**,**b**) Show representative images of LVPN dendritic arbors from WT and TDP-43^Q331K^ LVPNs, respectively. (**c**,**d**) Show representative images of the apical dendritic shaft and projecting dendritic spines in WT and TDP-43^Q331K^ LVPNs, respectively. (**e**,**f**) Show representative images of the basal dendritic shaft and projecting dendritic spines in WT and TDP-43^Q331K^ LVPNs respectively. (**g**) Shows a scatterplot with mean ± s.e.m. of the unchanged total dendritic arbor (μm) in TDP-43^Q331K^ LVPNs (*n* = 11) compared to WT age- and litter-matched controls (*n* = 12), Students unpaired two-tailed *t* test. (**h**) Shows a scatterplot with mean ± s.e.m. of the unchanged apical dendritic arbor (μm) in TDP-43^Q331K^ LVPNs (*n* = 11) compared to WT age- and litter-matched controls (*n* = 12), Students unpaired two-tailed *t* test. (**i**) Shows a scatterplot with mean ± s.e.m. of the increased apical spine density (spines per 100 μm of dendrite) in TDP-43^Q331K^ LVPNs (*n* = 11) compared to WT age- and litter-matched controls (*n* = 12), *P < 0.05, Students unpaired two-tailed *t* test. (**j**) Shows a scatterplot with mean ± s.e.m. of the increased basal spine density (spines per 100 μm of dendrite) in TDP-43^Q331K^ LVPNs (*n* = 11) compared to WT age- and litter-matched controls (*n* = 12), *P < 0.05, Students unpaired two-tailed *t* test. Scale bars: (**a**,**b**) represents 100 μm; (**c**–**f**) represents 5 μm.

Morphological properties of LVPNs from the motor cortex remained stable in TDP-43^Q331K^ mice, compared to WT age- and litter-matched controls. These measurements included: somatic volume, the total arbor length (the sum of the apical and basal dendritic lengths), the apical arbor length, the maximum apical terminal length (the distance from the soma to the apical dendritic termination closest to the edge of the cortical pia), the basal arbor length (the sum of all of the basal dendritic trees emanating from the soma), and the mean basal dendritic tree length (the mean length of each individual basal dendrite emanating from the soma; Table 2, Fig. 2). In addition to these gross morphological parameters, we examined apical and basal dendritic arbors with regard to their mean dendritic length per branch order, mean number of dendritic segments per branch order, and maximum branch ramifications. This allows for a finer degree of assessment than the above measures^31^,^32^. There were no significant changes in the mean dendritic length per branch order (*P* = 0.90), mean number of dendritic segments per branch order (*P* = 0.46), and maximum branch ramifications (*P* = 0.78) of LVPN apical dendrites of TDP-43^Q331K^ mice compared to age- and litter-matched WT controls. There was also no significant change in the mean dendritic length per branch order (*P* = 0.17), mean number of dendritic segments per branch order (*P* = 0.24) and maximum branch ramifications (*P* = 0.39) of LVPN basal dendrites of TDP-43^Q331K^ mice compared to age- and litter-matched WT controls.

The increase in frequency of EPSCs could be due to an increase in the number of presynaptic inputs onto LVPNs within the motor cortex. As dendritic spines are a morphological proxy of excitatory synapses in pyramidal neurons^33^,^34^, we quantified the dendritic spine densities (spines per 100 μm of dendrite) of motor cortex LVPNs from both WT and TDP-43^Q331K^ mice. By contrast to the maintenance of dendritic morphology, the total spine density of LVPNs of the motor cortex was increased by 53% in TDP-43^Q331K^ mice compared to age- and litter-matched WT controls (*P = 0.02; Table 2). This increase in spine density was found in both proximal and distal compartments of apical and basal dendrites of LVPNs within the motor cortex. Compared to WT controls, TDP-43^Q331K^ mice presented an increase of 48% in the total apical spine density (*P = 0.03; Table 2, Fig. 2), 57% in the total apical proximal spine density (1^st^ and 2^nd^ order branch segments; *P = 0.04; Table 2), 58% in the total apical distal spine density (3^rd^ order and beyond branch segments; **P = 0.006; Table 2). Similarly, compared to WT controls TDP-43^Q331K^ mice presented an increase of 55% in the total basal spine density (*P = 0.03; Table 2, Fig. 2), 75% in the total basal proximal spine density (1^st^ and 2^nd^ order branch segments; **P = 0.004; Table 2), and 53% in the total basal distal spine density (3^rd^ order and beyond branch segments; *P = 0.03; Table 2).

**Figure 3:**
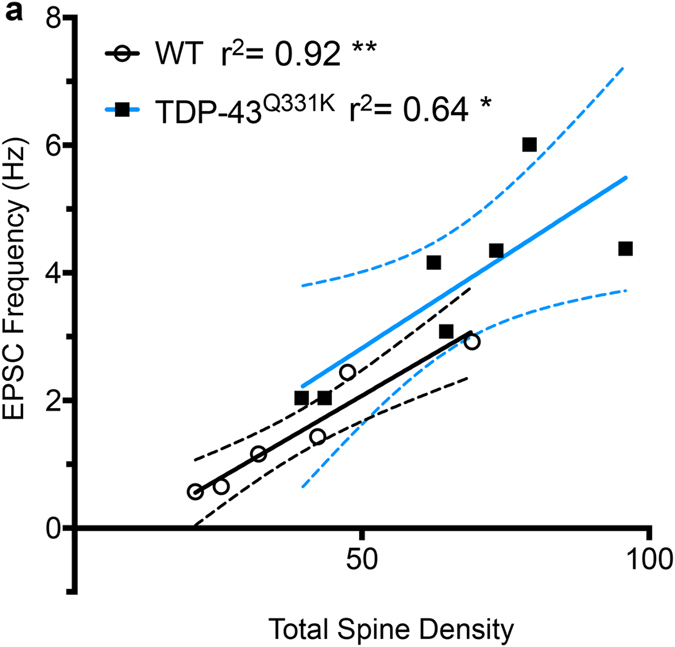
Correlation and linear relationship of spontaneous EPSC and total spine density of LVPNs from TDP-43^Q331K^ and WT controls. (**a**) Shows WT (open circles) and TDP-43^Q331K^ (black squares) LVPNs that have both recorded spontaneous EPSCs and had their dendritic arbors assessed for total spine density. These variables were highly correlated (Pearson’s *r*^2^; WT: 0.92, **P = 0.002; TDP-43^Q331K^: 0.64, *P = 0.03). Linear regression revealed no changes in slope between WT (black line) and TDP-43^Q331K^ (blue line), with hashed lines indicating respective 95% confidence intervals (*P* = 0.82). A runs test indicated that there were no departures from linearity in either WT (*P* > 0.99) nor TDP-43Q^331K^ (*P* = 0.80).

Using morphology alone, we are unable to identify if increased spines are functional or silent. We therefore correlated total spine density and EPSC frequency in the subset of LVPNs where we recorded spontaneous synaptic currents and subsequently recovered dendritic morphology (Fig. 3). In both WT and TDP-43^Q331K^ LVPNs, EPSC frequency was highly correlated with total spine density (WT: *r*^2^ = 0.92, **P = 0.002; TDP-43^Q331K^: *r*^2^ = 0.64, **P = 0.03). In addition, regression analysis revealed a linear relationship between the spontaneous EPSC frequency and spine density, with the slope unchanged between WT and TDP-43^Q331K^ LVPNs (WT: slope = 0.052 ± 0.008, *n* = 6; TDP-43^Q331K^: slope = 0.058 ± 0.019, *n* = 7, *P* = 0.82). A runs test showed that there was no deviation from linearity in this relationship between either genotype (WT: *P* > 0.99; TDP-43^Q331K^: *P* = 0.80). Collectively, this data suggests that the proportion of functional compared to silent spines or synapses is unchanged between WT and TDP-43^Q331K^ and that the increased spine densities evidenced in TDP-43^Q331K^ LVPNs are likely contributing to the observed increase in spontaneous EPSC frequency.

### Spine density increases in TDP-43^Q331K^ LVPNs remain conserved regardless of neuronal subtype

**Figure 4:**
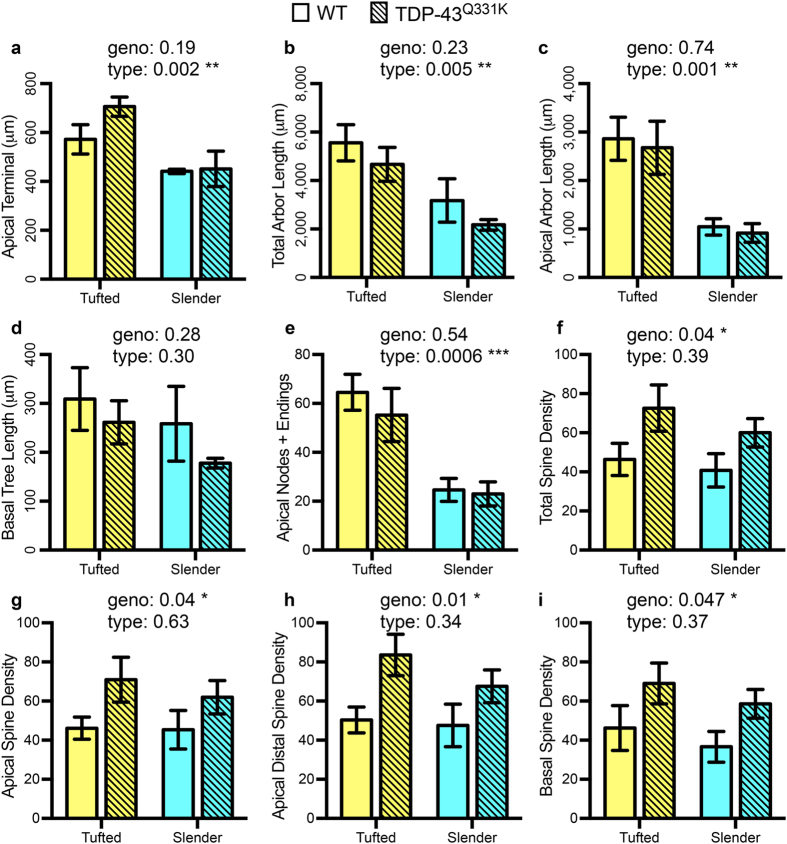
Dendritic length characteristics may be stratified according to tufted or slender neuronal types, however spine density characteristics are only affected by genotype. WT (not hashed) and TDP-43^Q331K^ (hashed) LVPNs were classified into tufted and slender pyramidal neurons. Compared to tufted LVPNs, slender LVPNS are significantly reduced in length in their apical terminal length (**a**), total arbor length (**b**) and apical arbor length (**c**), with no significant difference between WT controls and TDP-43^Q331K^ LVPNs of either type. There was no difference based on neuronal type or genotype in the mean basal tree length of LVPNs (**d**). Compared to tufted LVPNs, slender LVPNS are significantly reduced in the number of apical bifurcation nodes and dendritic endings (**e**), with no significant difference between WT controls and TDP-43^Q331K^ LVPNs of either type. Compared to TDP-43^Q331K^ LVPNs, WT LVPNs had reduced total spine density (**f**), apical spine density (**g**), apical distal spine density (**h**) and basal spine density (**i**), with no difference between tufted and slender neuronal types. All data represented as mean ± s.e.m., two-way ANOVAs (tufted: *n* = 7 WT and TDP-43^Q331K^; slender: WT *n* = 5 WT, *n* = 4 TDP-43^Q331K^), *P < 0.05, **P < 0.01, ***P < 0.001.

Cortical pyramidal neurons consist of many morphological and functional subtypes^35^,^36^,^37^, with motor cortex LVPNs projecting to different targets both cortically and subcortically^35^,^36^,^37^. Based on established morphological criteria, such as soma length and distal apical branching^38^, we classified our LVPNs into two major classes: tufted and slender. The overall trend of unchanged dendritic length characteristics (maximum apical terminal length (μm), total dendrite length (μm), mean apical tree length (μm) and mean basal tree length (μm)) between TDP-43^Q331K^ LVPNs and age- and litter-matched WT controls was conserved in this comparison, with expected length restrictions in total and apical dendrites based on less elaborate distal trees (Table 3, Fig. 4).

The amount of apical branching was increased in tufted compared to slender LVPN, as indicated by the total number of bifurcation nodes and terminal endings. There was no difference in the total number of bifurcation nodes and terminal endings in basal arbors of tufted and slender LVPNs (Table 3, Fig. 4). No branching alterations were apparent when comparing WT and TDP-43^Q331K^ LVPNs (Table 3).

When considering spine density (total spine density, apical spine density, apical distal spine density and basal spine density), there was an increased spine density of both tufted and slender TDP-43^Q331K^ LVPNs compared to age- and litter-matched controls (Table 3, Fig. 3). There was no significant difference between the spine densities of tufted and slender neuronal types (Table 3, Fig. 4). Overall, our data suggests that the spine density changes observed between all filled TDP-43^Q331K^ and WT LVPNs (Table 2, Fig. 2) are recapitulated when we stratify our data based on morphological classification of tufted and slender LVPNs (Table 3, Fig. 4).

### There are no sex differnces in LVPNs from TDP-43^Q331K^ and WT

**Figure 5:**
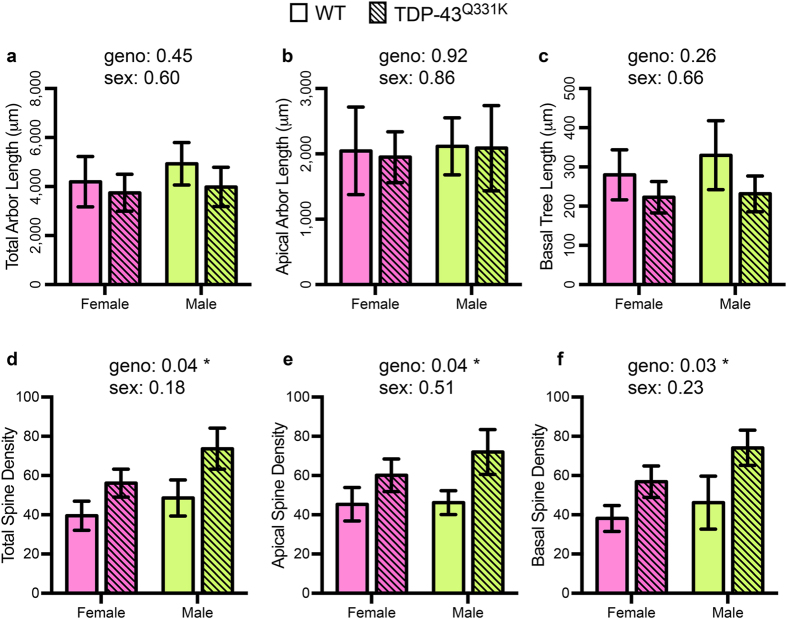
No observed sex differences in morphological characteristics of motor cortex LVPNs from TDP-43^Q331K^ and WT mice. WT (not hashed) and TDP-43^Q331K^ (hashed) LVPNs were divided into groups based on gender. LVPN dendritic lengths are unchanged in total arbor length (**a**),apical arbor length (**b**) and mean basal tree length of LVPNs (**c**), with no significant difference between WT controls and TDP-43^Q331K^ LVPNs of either genotype or sex. Compared to WT LVPNs, TDP-43^Q331K^ LVPNs had increased total spine density (**d**), apical spine density (**e**), apical distal spine density (**f**) and basal spine density (**i**), with no difference between sexes. All data represented as mean ± s.e.m., two-way ANOVAs (female: *n* = 6 WT, *n* = 4 TDP-43^Q331K^; male: WT *n* = 6 WT, *n* = 7 TDP-43^Q331K^), *P < 0.05.

TDP-43 mutations are known to exhibit sex dimorphisms in rodent models^39^, and the sex of ALS patients influences the incidence and clinical features^40^. We therefore analysed our morphologic parameters with groups divided according to both sex and genotype. These parameters were total arbor length, apical arbor length, mean basal tree length, total spine density, apical spine density and basal spine density (Table 4, Fig. 5). We observed no changes in dendritic length characteristics (total arbor length, apical arbor length and mean basal tree length) of motor cortex LVPNs between either sex or between TDP-43^Q331K^ and age- and litter-matched WT controls (Table 4, Fig. 5). Regarding spine density (total spine density, apical spine density and basal spine density), there was an increased spine density of both male and female TDP-43^Q331K^ LVPNs compared to age- and litter-matched controls, however there were no significant differences between the spine densities of either sex (Table 4, Fig. 5). When considering male and female groups as discreet experiments, there was no difference between gender in the calculated effect sizes of genotype for total spine density (Cohen’s *d*, female: 1.02; male: 1.00) and basal spine density (Cohen’s *d*, female: 1.16; male: 0.97), which were both, by convention, large effect sizes. By contrast, the calculated effect size of genotype for apical spine density was large in males (Cohen’s *d*, male: 1.08) and medium in females (Cohen’s *d*, female: 0.78). These data suggest that the spine density changes observed between all filled TDP-43^Q331K^ and WT LVPNs (Table 2, Fig. 2) are conserved with respect to sex, and that sex itself has little bearing on LVPN morphology nor spine density (Table 4, Fig. 5).

## Discussion

Degeneration and eventual loss of both upper and lower MNs is a hallmark for ALS. Our understanding of the cortical contribution to the pathogenesis of ALS has increased in recent years, with multiple lines of evidence suggesting that early synaptic hyperexcitability^16^,^17^,^18^,^21^,^22^,^23^,^41^,^42^,^43^,^44^,^45^ (either intrinsic, or by glutamatergic synaptic input increase or GABAergic inhibitory deficits) precedes the cellular degeneration and later neuro-motor system neurotransmission failures, supporting earlier work in the field^9^,^11^,^15^,^46^,^47^,^48^.

Here, we report for the first time that increased excitatory synaptic inputs and synaptic drive ratio onto LVPNs occurs in the TDP-43^Q331K^ mouse model of ALS, in conjunction with increased dendritic spine densities of apical and basal dendritic arbors. These findings are consistent with previous reports of morphological changes in TDP-43^A315T^ models, as well as functional and morphological changes in SOD1^G93A^ models, including early synaptic excitatory/inhibitory input abnormalities^16^,^17^,^45^ and spine density alterations^16^,^21^,^22^,^45^, preceding the regression of the dendritic arbor and eventual loss of LVPNs within the motor cortex^16^,^21^,^22^,^26^,^43^,^45^,^49^. However, some key differences exist between our findings in TDP-43^Q331K^ mice and those previously reported for SOD1^G93A^ and TDP-43^A315T^ mice. In line with clinical studies^9^,^10^,^11^,^12^,^42^,^46^,^48^, increased excitatory neurotransmission may be a key pathogenic mechanism that has been recapitulated functionally in SOD1^G93A^ rodent models^16^,^17^. Although impaired inhibitory neurotransmission and dendritic arbor regression has been previously reported in TDP-43^A315T^ mice, these changes were not accompanied by increased excitatory synaptic transmission or increased dendritic spine density^45^; however, subsequent investigations, without reporting inhibitory synaptic transmission, have reported decreased excitatory synaptic transmission by P60^50^. Our electrophysiological measurements of spontaneous EPSCs and IPSCs consist of a combination of miniature (TTX insensitive) synaptic currents and synaptic currents caused by presynaptic neuron firing (TTX sensitive) within our *in vitro* cortical slice preparations. Although our results may have been influenced by presynaptic release probability and presynaptic activity, similarly aged SOD1^G93A^ mice, increased spontaneous EPSC frequency was matched by increased miniature EPSC frequency, compared to WT^17^. In other recordings of cortical pyramidal neurons where synaptic drive ratio was measured, spontaneous and miniature synaptic drive ratios were both increased^51^.

Here, we have analysed spine density in proximal and distal compartments of both apical and basal dendrites, and found increases in spine density that correlate strongly with established mechanisms of synaptic plasticity^33^,^34^,^52^. The increases in spine density of TDP-43^Q331K^ LVPNs compared to WT controls remain significant, whether we stratify neurons based on neuronal type (tufted or slender) or based on sex. In addition, the relationship between spontaneous EPSC frequency and total spine density is linear, indicating the proportion of immature or silent to active spines remains comparable between both genotypes. This is consistent with an activity-responsive role for TDP-43, where depolarization causes TDP-43 relocation to dendrites and dendritic spines, where it regulates local translation^53^.

Intriguingly, the increased spine density seen in LVPNs of TDP-43^Q331K^ mice contrasts markedly with the decrease in spine density seen in similarly aged SOD1^G93A^ mice^16^,^21^, which show a far more aggressive neuronal loss within the cortex and other brain areas^26^,^43^,^54^. We propose that the substantial delay in neuronal loss in the cortex of P26-35 TDP-43^Q311K^ mice (compared to the SOD1^G93A^ model) is indicative of a much earlier presymptomatic stage in this model, when compared to P26-35 SOD1^G93A^ mice, which have already entered a period of dendritic and dendritic spine degeneration^16^,^21^. Indeed a recent study in TDP-43^A315T^ mice showed decreased spine density by P60^50^, substantially later than the earliest findings in the SOD1 model^16^,^21^. A similar progression pattern, with neurons exhibiting increased spine density compared to controls, followed by a lower spine density compared to controls at late stages of disease, has been reported for Huntington’s disease patients^55^,^56^.

Although we are sampling from a neuronal population within the motor cortex that contains LVPNs projecting to targets other than the spinal cord^22^,^35^,^43^,^57^, previous work has shown that over 45% of the LVPNs within this region are indeed cortico-spinal^58^. Cortico-spinal LVPNs (upper MNs), along with lower brainstem and spinal cord MNs, are particularly vulnerable in ALS and exhibit increased EPSCs in rodent models^16^,^17^,^18^. It is important to note that studies of motor cortex pyramidal neurons in *post mortem* human tissue are similarly unable to distinguish between corticospinal neurons and other large pyramidal neurons with different cortical or sub-cortical targets^15^,^59^ and only 1–2% of pyramidal tract projection fibres are from these cortico-spinal LVPNs^60^. Furthermore, volumetric imaging assessments of cortical volumes of ALS patients also have this limitation. Indeed, current results place ALS and related conditions on a clinical continuum with fronto-temporal dementia, based on the widespread cerebral pathologic and genetic associations between the two conditions^1^,^4^,^61^,^62^.

Our classification of neurons into tufted and slender has implications for the likely projection targets of the labelled LVPN, with tufted (Type I) LVPNs more likely to be projecting sub-cerebrally, and slender (Type II) LVPNs likely to be targeting intra-columnar, inter-hemisphere (callosal) or ipsilateral cortical areas^38^,^63^. Although our classification system is limited compared to more recent molecular (notably ctip2 expression ref. 64) and functional approaches, (see refs 35, 36, 37 for reviews), our observations of increased spine density in TDP-43^Q331K^ LVPNs compared to WT, when accounting for neuronal type (tufted and slender) ties into a broadening of ALS clinical features to include callosal and further cerebral involvement^65^,^66^,^67^ and is in line with widespread (regional and layer) cortical changes in rodent models^21^,^50^.

Despite the absence of any robust effect of gender on our observations, we did find subtle changes in the effect size WT and TDP-43^Q331K^ between the sexes, with males having a greater increase in apical spine density compared to females. Increased effects of genetic factors in males may underlie the increased risk of cognitive impairment in male ALS^68^. Further investigation of gender dimorphisms on neuronal structure and function in ALS are warranted and may uncover important differences that may underlie disease processes^40^ and influence diagnosis^69^.

In summary, our study demonstrates that increased spontaneous excitatory synaptic inputs and increased dendritic spine density are evident in TDP-43^Q331K^ LVPNs of the motor cortex from P26-35, long before these neurons exhibit gliosis and death at 24 months^26^. These dendritic spines and electrophysiological findings are consistent with clinical studies^10^,^12^,^46^,^70^ and this rodent model displays a TDP-43 inclusion pathology^26^ that mirrors a large proportion of the sporadic clinical population^6^,^7^. Understanding how these abnormalities arise, assaying underlying intrinsic firing properties and determining if they are also present in lower MNs, will be of key importance if discoveries from TDP-43 models are to have translational theraputic success.

## Methods

### Ethics Statement

All experimental procedures were approved by The University of Queensland Animal Ethics Committee and complied with the policies and regulations regarding animal experimentation and other ethical matters, in accordance with the Queensland Government Animal Research Act 2001, associated Animal Care and Protection Regulations (2002 and 2008), as well as the Australian Code for the Care and Use of Animals for Scientific Purposes, 8th Edition (National Health and Medical Research Council, 2013).

### Mice

Wild-type (WT) and TDP-43^Q311K^ mice were bred at the University of Queensland in an identical manner to past studies^26^,^49^. Genotyping was carried out as previously described^26^,^49^. Experiments were conducted on age and litter-matched mice between P26-P35. A total of 10 WT (7 male, 3 female) and 6 TDP-43^Q311K^ (4 males, 2 female) from 5 litters with no difference in the ratio of males to females between genotypes were used in this study. For electrophysiology, recordings from 8 WT (5 male, 3 female) and 6 TDP-43^Q311K^ (4 males, 2 female) were included in this data.

### Slice preparations

Mice were sacrificed by sodium pentobarbitone overdose (60–80 mg/kg, i.p. Vetcare, Brisbane, Australia) and then decapitated. The brain was quickly removed and bathed in ice-cold high-Mg^2+^ Ringer solution, that contained, (in mM): 130 NaCl, 3 KCl, 26 NaHCO_3_, 1.25 NaH_2_PO_4_, 5 MgCl_2_, 1 CaCl_2_, and 10 D-glucose (osmolarity ~300 mOsm, Vapro 5520 osmometer, Wescor, South Logan, UT)^71^. Ringer solution was continuously bubbled with 95% O_2_/5% CO_2_ to maintain pH at 7.4. Off-coronal sections (300 μm thick) containing the motor cortex were cut using a vibratome (Leica VT 1200 S, Leica Biosystems) in a manner identical to prior studies, using the aid of a brain atlas^16^,^21^,^72^. Cut slices were transferred from ice-cold high-Mg^2+^ Ringer solution and incubated for 60 min in the same solution warmed to 34 °C in a water bath. The sections were transferred to a normal Ringer solution (1 mM MgCl_2_, 2 mM CaCl_2_) prior to start of recording and labelling.

### Electrophysiology

Patch electrodes were pulled from borosilicate glass capillaries (Vitrex Modulohm, Edwards Medical, NSW Australia) giving a tip resistance of ~3–4 MΩ. The tip of the electrode was filled with 2% Neurobiotin (NB; Vector Labs, Burlingame CA, USA) in an artificial intracellular solution containing (in mM): 135 mM Cs^+^-methanesulphonate (Cs^+^MeSO_4_), 6 mM KCl, 1 mM EGTA (ethylene glycol bis (2-aminoethyl ether)-N,N,N′,N′-tetraacetic acid), 2 mM MgCl_2_, and 5 mM Na-HEPES (Na–4-2-hydroxyethyl-1-piperazineethanesulfonic acid), 3 mM ATP-Mg^2+^, 0.3 mM GTP-Tris (pH 7.25 with KOH, osmolarity of 305 ± 5 mOsm)^16^. The electrode was then backfilled with the same intracellular solution without NB. A single brain slice was placed in a tissue chamber on a Nikon E600FN (Tokyo, Japan) microscope fitted with IR-DIC optics, and continuously superfused (1–2 ml/min) with the normal Ringer solution at room temperature (22 to 24 °C). The tissue was stabilized with the aid of a metal mesh and recording electrodes were viewed on a monitor through a 60×/1.3NA water-immersion objective and infrared video camera (Hamamatsu, Japan). Recordings and voltage pulse protocols were made with an Axopatch 1D amplifier (Axon Instruments, Foster City, CA, USA) and adapted from procedures developed in the brainstem^31^. Data were acquired at a sampling rate of 10 kHz, low-pass filtered at 2 kHz using PClamp 8.2 software and a Digidata 1332 A digitizer (Axon Instruments). Individual LVPNs within the motor cortex were targeted visually and a patch-electrode was advanced towards the cell body using a micromanipulator (MPC-200, Sutter Instrument Company). The electrode tip was placed against the neuronal soma and gentle suction was applied until a stable seal of 1 GΩ (tight-seal) was obtained. Square-wave voltage steps (0.5 s, 1 Hz) of 5–25 mV that generated current pulses of 300–500 pA were applied until rupturing of the membrane occurred. As soon as the membrane broke, pulses were stopped, and spontaneous EPSCs and IPSCs were recorded at holding membrane potentials of −70 mV and 0 mV, respectively. Synaptic currents were recorded in 2 min epochs and analyzed offline using PClamp 10.2. Between 400–1500 synaptic events were analysed from each cell to determine electrophysiological properties. Following the recording of synaptic currents, square wave pulses were applied for 3–6 minutes to electroporate NB into the neuron. Slices were then left for approximately 5 minutes to allow for NB diffusion, then fixed in 4% paraformaldehyde in 0.1 M phosphate buffered saline (PBS), pH 7.4, for 30 minutes, washed in PBS, and incubated for 4 hours in PBS containing 4% bovine serum albumin (BSA) and 0.5% Triton X-100 at 4 °C. Slices were then incubated for 4 hours at 4 °C in Cy3-streptavidin (Sigma; 1:500 in 4% BSA in PBS) to visualize NB. Slices were washed in PBS and mounted on glass slides in a standard glycerol-based *p*-phenylenediamine mounting medium and cover-slipped.

### Imaging

Morphological properties of the LVPNs from the motor cortex filled with NB were analyzed from stacks of confocal images obtained at 63X magnification as described previously^16^. Images containing a mosaic of the total dendritic arbors were acquired on a Leica TCS SP8 confocal microscope using a Leica NA 63×/1.3NA glycerol immersion objective with a voxel size of 0.18 × 0.18 × 0.5 μm/pixel (x, y and z-dimensions). Image z-stacks of between 100 and 180 images were acquired at a z-separation of 0.5 μm. For spine quantification, we obtained images with the same objective at 2.5 zoom. The voxel size for spine images was 0.07 × 0.07 × 0.33 μm/pixel (x, y and z-dimensions). Image z-stacks of dendrites contained between 25 to 135 images acquired at a step size of 0.33 μm. A total of 96,207 μm of dendritic arbor was traced for the neuron morphology component of this study.

### Morphological analysis

Morphological properties of NB-filled LVPNs from the motor cortex were analysed using Neurolucida (MBF Bioscience Inc, Williston, VA, USA) with manual soma, dendrite and dendritic spine tracing in a manner identical to previous studies of LVPNs within the motor cortex^16^. Dendritic processes were classified as spines only if they were <3 μm long and <0.8 μm in cross-sectional diameter, in accordance with past studies^16^,^52^. Neurons included for analysis must have shown exceptional filling and contained an intact basal or apical (or both) dendritic arbors. An arbor consisted of the entirety of the length of the dendritic trees emanating from the neuronal soma. A dendritic tree consisted of all of the branches emanating from a single primary (1^st^ order) branch extending from the neuronal soma^31^. Proximal and distal dendrites were classified according to centrifugal branch orders, where branches extending from the cell soma are considered first order, branch ramifications from this dendritic segment second order, and ramifications from these segments being classified as third order^31^. Proximal dendrites consisted of first- and second-order branches, whereas distal dendrites consisted of third-order branches and beyond, as previously established^31^. Neurons were subsequently categorised based on classical slender or thick-tufted morphological criteria^38^.

### Statistical Methods

Mean and standard error of the mean (s.e.m.) were calculated for each data set. For the purposes of this study, each individual LVPN recorded from and/or morphologically assessed is considered the *n*, in accordance with other neurophysiological studies in the field^16^,^18^. From previous work with synaptic currents, we calculated an expected effect size, Cohen’s *d*^73^ based on past results using ALS rodent models^16^,^18^. Using GPower 3 software^74^ and our expected effect size (1.6), alpha (0.05) and beta (0.8) our required *a priori* minimum sample size was *n* = 7 per group. Sample sizes were also commensurate with those used in previous studies. All statistical tests were calculated with Prism 7 (Graphpad, San Diego, CA). If groups displayed normal distributions and no difference in their variances, unpaired two tailed *t*-tests were used, otherwise a non-parametric Mann Whitney test was used as indicated. For all branch order analysis, two-way ANOVAs were used with genotype and branch order the factors. All correlations were performed with Pearson’s coefficients. Statistical significance was accepted at *P* < 0.05. All data is presented as means ± s.e.m. All animal experiments were performed in a randomized manner (mice allocated based on random order of litter tail-tipping for genotyping) and all analysis was performed blind to genotype.

## Additional Information

**How to cite this article**: Fogarty, M. J. *et al*. Cortical synaptic and dendritic spine abnormalities in a presymptomatic TDP-43 model of amyotrophic lateral sclerosis. *Sci. Rep.* **6**, 37968; doi: 10.1038/srep37968 (2016).

**Publisher's note:** Springer Nature remains neutral with regard to jurisdictional claims in published maps and institutional affiliations.

## Figures and Tables

**Table 1:** EPSC and IPSC parameters of LVPNs within the motor cortex.

Parameter	WT (*n* = 11)	TDP-43^Q311K^ (*n* = 10)	*P* value
EPSC frequency (Hz)	1.48 ± 0.23	3.67 ± 0.50	**0.001^a^
EPSC amplitude (pA)	−9.89 ± 1.86	−7.55 ± 1.96	0.40
EPSC rise time (ms)	3.65 ± 0.37	3.67 ± 0.49	0.97
EPSC half-width (ms)	2.93 ± 0.38	2.13 ± 0.35	0.14
IPSC frequency (Hz)	3.19 ± 0.48	4.38 ± 0.64	0.15
IPSC amplitude (pA)	25.02 ± 5.59	11.30 ± 6.51	0.12
IPSC rise time (ms)	7.31 ± 1.94	9.32 ± 1.30	0.40
IPSC half-width (ms)	7.80 ± 1.71	10.88 ± 3.12	0.39
Synaptic drive ratio (E/I)	0.52 ± 0.09	0.92 ± 0.12	*0.01

All data presented as mean ± s.e.m., number of cells (*n*) in parenthesis. ^*^P < 0.05, ^**^P < 0.01, Student’s two-tailed unpaired *t*-test, except^a^, Mann Whitney test.

**Table 2:** General morphological parameters and dendritic spine density (spines per 100 μm of dendrite) of LVPNs within the motor cortex.

Parameter	WT (*n* = 12)	TDP-43^Q311K^ (*n* = 11)	*P* value
Soma volume (μm^3^)	2,546 ± 307	2,241 ± 300	0.49
Total dendrite length (μm)	4,566 ± 651	3,765 ± 581	0.37
Mean apical tree length (μm)	2,082 ± 381	2,038 ± 436	0.94
Max apical terminal length (μm)	517.9 ± 39.3	613.5 ± 51.5	0.15
Total basal tree length (μm)	2,410 ± 338	1,727 ± 173	0.19^a^
Mean basal tree length (μm)	288.1 ± 47.4	228.4 ± 31.1	0.31
Total spine density	44.1 ± 5.8	67.3 ± 7.4	*0.02
Total apical spine density	45.8 ± 5.0	67.7 ± 7.8	*0.03
Proximal apical spine density	28.4 ± 3.9	44.5 ± 6.4	*0.04
Distal apical spine density	49.2 ± 5.7	77.7 ± 7.5	**0.006
Total basal spine density	42.2 ± 7.3	65.3 ± 7.1	*0.03
Proximal basal spine density	31.8 ± 3.8	55.5 ± 6.6	**0.004
Distal basal spine density	47.4 ± 8.4	72.4 ± 7.0	*0.03

All data presented as mean ± s.e.m., number of cells (*n*) in parenthesis. ^*^P < 0.05, ^**^P < 0.01, Student’s two-tailed unpaired *t*-test, except^a^, Mann Whitney test.

**Table 3:** Dendritic morphology of tufted and slender LVPNs within the motor cortex in TDP-43^Q331K^ compared to WT.

Parameter	Tufted (*n* = *7*)	Slender (*n*)	ANOVA *P*
Max apical terminal length (μm)	WT: 573 ± 60 TDP-43: 3,751 ± 750	WT: 441 ± 9 (5) TDP-43: 451 ± 73 (4)	Type: *P* = 0.002 **Geno: *P* = 0.18
Total dendrite length (μm)	WT: 5,559 ± 746 TDP-43: 4,675 ± 700	WT: 3,177 ± 895 (5) TDP-43: 2,173 ± 221 (4)	Type: *P* = 0.005 **Geno: *P* = 0.23
Mean apical tree length (μm)	WT: 2,862 ± 444 TDP-43: 2,677 ± 548	WT: 1,045 ± 168 (5) TDP-43: 918 ± 192 (4)	Type: *P* = 0.001 **Geno: *P* = 0.74
Mean basal tree length (μm)	WT: 309 ± 64 TDP-43: 262 ± 44	WT: 258 ± 77 (5) TDP-43: 178 ± 10 (4)	Type: *P* = 0.28 Geno: *P* = 0.30
Apical nodes & endings	WT: 65 ± 7.4 TDP-43: 55 ± 11	WT: 25 ± 4.7 (5) TDP-43: 23 ± 4.9 (4)	Type: *P* < 0.001 ***Geno: *P* = 0.54
Basal nodes & endings	WT: 47 ± 6.6 TDP-43: 46 ± 11	WT: 40 ± 11 (5) TDP-43: 31 ± 3.4 (4)	Type: *P* = 0.20 Geno: *P* = 0.58
Total spine density	WT: 46.4 ± 8.2 TDP-43: 72.6 ± 11.9	WT: 40.8 ± 8.5 (5) TDP-43: 60 ± 7.7 (4)	Type: *P* = 0.39 Geno: *P* = 0.04*
Apical spine density	WT: 46.1 ± 5.7 TDP-43: 70.9 ± 11.5	WT: 45.4 ± 9.9 (5) TDP-43: 62 ± 8.5 (4)	Type: *P* = 0.62 Geno: *P* = 0.04*
Apical distal spine density	WT: 50.3 ± 6.6 TDP-43: 83.6 ± 10.6	WT: 47.5 ± 10.9 (5) TDP-43: 67.6 ± 8.3 (4)	Type: *P* = 0.34 Geno: *P* = 0.01*
Basal spine density	WT: 45.8 ± 11.2 TDP-43: 69 ± 10.6	WT: 36.6 ± 7.8 (5) TDP-43: 58.6 ± 7.3 (4)	Type: *P* = 0.37 Geno: *P* = 0.04*

All data presented as mean ± s.e.m., number of cells (*n*) in parenthesis. All analysis are two-way ANOVAs for neuronal subtype and genotype where ^*^P < 0.05. Bonferroni post-tests showed were all *P* > 0.05.

**Table 4:** No significant sex differences of general morphologic parameters and dendritic spine density (spines per 100 μm of dendrite) of LVPNs within the motor cortex.

Parameter	Female (*n*)	Male (*n*)	ANOVA *P*
Total dendrite length (μm)	WT: 4,201 ± 1,028 (6) TDP-43: 3,751 ± 750 (4)	WT: 4,932 ± 869 (6) TDP-43: 3,986 ± 800 (7)	Sex: *P* = 0.60 Geno: *P* = 0.46
Mean apical tree length (μm)	WT: 2,047 ± 670 (6) TDP-43: 1,950 ± 435 (4)	WT: 2,116 ± 869 (6) TDP-43: 2,089 ± 652 (7)	Sex: *P* = 0.86 Geno: *P* = 0.92
Mean basal tree length (μm)	WT: 280 ± 64 (6) TDP-43: 223 ± 40 (4)	WT: 330 ± 88 (6) TDP-43: 232 ± 46 (7)	Sex: *P* = 0.66 Geno: *P* = 0.26
Total spine density	WT: 39.5 ± 7.4 (6) TDP-43: 56.2 ± 7.2 (4)	WT: 48.6 ± 9.1 (6) TDP-43: 73.7 ± 10.5 (7)	Sex: *P* = 0.18 Geno: *P = 0.04
Apical spine density	WT: 45.4 ± 8.5 (6) TDP-43: 60.2 ± 8.4 (4)	WT: 46.3 ± 8.4 (6) TDP-43: 72.0 ± 11.4 (7)	Sex: *P* = 0.51 Geno: *P = 0.04
Basal spine density	WT: 38.2 ± 6.6 (6) TDP-43: 56.9 ± 8.0 (4)	WT: 48.2 ± 13.6 (6) TDP-43: 74.1 ± 9.0 (7)	Sex: *P* = 0.23 Geno: *P = 0.03

All data presented as mean ± s.e.m., number of cells (*n*) in parenthesis. All analysis are two-way ANOVAs for sex and genotype where ^*^P < 0.05. Bonferroni post-tests showed were all *P* > 0.05.
